# The Animal Parasites of Sheep

**Published:** 1890-08

**Authors:** 


### REVIEW DEPARTMENT.

THE ANIMAL PARASITES OF SHEEP. By COOPER CURTICE, D.V.S., M.D. Published by the United States Department of Agriculture, Bureau of Animal Industry.

It is with pleasure that we note the new departure of the Bureau of Animal Industry as instanced by Dr. Curtice's very exhaustive study of the animal parasites of the sheep. Bacteriology has occupied the scientific public so extensively for a decade of years that other older, yet hardly less important, branches of pathological investigation have been neglected and almost lost sight of. The results obtained from our researches into the etiology of the infectious diseases have been so astounding, and so pregnant of promise for the future, that it has almost obtained our exclusive attention.

The treatise is a systematic one, and discusses the various parasites that affect the sheep. A history of the discovery of the parasite is given, followed by description, life history, occurrence, disease caused by it, and concluding with a section on treatment. In a number of cases detailed results of experiments made at the agricultural station add a certain amount of original material to our stock of knowledge concerning these parasites. Thirty-six plates, lithographic and chromo-lithographic, illustrate the volume. Their execution is good, though the author does not tell us how many of them are the result of original study, and how many should be credited to preceding investigators.

After some remarks on ovine parasitology in general, the author describes the variety of œstrus affecting the sheep, with three plates—one devoted to the appearance and anatomy of the mature and immature parasite, and two showing the larvæ *in situ* in the sinuses of the head. The so-called sheep tick and the sheep and goat louse are next considered, and are illustrated in Plates IV, V, and VI.

The important class of itch insects are next considered, and the author describes three varieties, viz.: Larcoptes scabiei (head scab), Psoroptes communis (common scab) and Chorioptes communis (foot scab). The first is probably identical with the ordinary human itch insect, or is a sub-variety of it; the last is very rare, and has only been seen in Germany. The author figures only the Psoroptes (Plates VII and VIII), which seems to be the only one that he has met with.

Of the Pentastamata only one variety, Linguatula tænioides, is mentioned. It is, apparently, very rare in this country.

The tapeworms are considered with great fulness. The anatomy of T. marginata is shown in Plate IX, and its embryos in the liver in Plate X. T. cœnurus (the cause of gid or staggers), T. echinococcus and its cysticerci, and T. tenella are then described and figured in Plate XI. The section on T. fimbriata and T. tenella contains the results of a number of valuable original investigations and experiments. Plates XII and XIII illustrate it. The same may be said of T. expansa (Plates XIV and XV).

The flukes, on account of the important ovine diseases they cause, might well have been given a larger space than the four or five pages which the author has accorded them. More especially is this the case since liver rot seems to be hardly recognized in America, though it must be not uncommon. In England it has been the source of immense losses to sheep raisers. Plate XVI illustrates the Distoma hepaticum, or common liver fluke, with the snail which is its intermediate host, the cercariæ, etc. Plate XVII shows Distoma lanceolatum and two closely-allied species of Amphistoma and Linguatula.

The round worm found in the stomach is the Strangylus contortus, Plate XVIII. Strangylus filicollis, Strangylus ventricosus, Ascaris lumbricoides, Dochinius cerumes and Sclerostoma hypostomma (Plates XIX, XX, XXI, XXII and XXIII) are those found in the intestines.

The author then describes at length, and figures in Plates XXIV to XXVII, a new nematode worm, which he calls the Æsophagostoma Columbraicum. It is a small round worm, some 15 mm. long, of which the adults inhabit the large intestine of sheep, while the embryos live in tumors in the intestinal walls, especially of the cæcal region. They are quite common in the sheep of the Southern and Southeastern portions of the United States, but seem to be unknown in Europe. They cause considerable mortality among the lambs and young sheep in the sections where they abound. Good illustrations of the parasite in its various stages, the intestinal tumors, etc., are given.

The variety of Trichocephalus affecting the sheep, T. affinis, is illustrated in Plate XXVIII.

The species of Strangylus which cause the various forms of verminous bronchitis and verminous pneumonia are next considered. It is probable the S. ovis pulmonalis, S. filaria and S. paradoxus may all cause these maladies. Plates XXIX and XXXI show the first-mentioned parasite, and Plates XXX and XXXII are excellent chromo-lithographs showing the appearance of the lung in sheep affected with this malady. Plate XXXIII shows micro-sections of lung with the parasite *in situ.* Plate XXXIV shows S. filaria, and Plates XXXV and XXXVI, in colors, show the lung as affected by it.

The author has thus described, in a very satisfactory manner, the varieties of parasites which affect our sheep, and has arranged his material in a systematic form, which renders it easy of reference and useful for all subsequent investigators in the same field. A bibliography and a fuller reference to preceding investigators would have added to its value. It is to be hoped that the parasites of the other domestic animals will soon be similarly described.

In conclusion, we would suggest that a little better print, paper and binding would be appropriate in a book of permanent value which is issued as a government report. W. S. G.

